# Cardiopulmonary Complications after Pulmonary Embolism in COVID-19

**DOI:** 10.3390/ijms25137270

**Published:** 2024-07-02

**Authors:** Carla Suarez-Castillejo, Néstor Calvo, Luminita Preda, Rocío Córdova Díaz, Nuria Toledo-Pons, Joaquín Martínez, Jaume Pons, Miquel Vives-Borràs, Pere Pericàs, Luisa Ramón, Amanda Iglesias, Laura Cànaves-Gómez, Jose Luis Valera Felices, Daniel Morell-García, Belén Núñez, Jaume Sauleda, Ernest Sala-Llinàs, Alberto Alonso-Fernández

**Affiliations:** 1 Servicio de Neumología, Hospital Universitario Son Espases, 07120 Palma de Mallorca, Spain; carla.suarez@ssib.es (C.S.-C.); ernest.sala@ssib.es (E.S.-L.); 2 Institut d’Investigació Sanitària Illes Balears (IdISBa), 07120 Palma de Mallorca, Spainpedroa.pericas@ssib.es (P.P.);; 3 Servicio de Radiodiagnóstico, Hospital Universitario Son Espases, 07120 Palma de Mallorca, Spain; 4 Servicio de Cardiología, Hospital Universitario Son Espases, 07120 Palma de Mallorca, Spain; 5 Facultad de Medicina, Universidad de las Islas Baleares, 07122 Palma, Spain; 6 Centro de Investigación Biomédica en Red de Enfermedades Respiratorias (CIBERES), Instituto de Salud Carlos III, 28029 Madrid, Spain; 7 Servicio de Análisis Clínicos, Hospital Universitario Son Espases, 07120 Palma de Mallorca, Spain

**Keywords:** COVID-19, SARS-CoV-2, pneumonia, thrombosis, pulmonary embolism, follow-up, cardiopulmonary complications

## Abstract

Although pulmonary embolism (PE) is a frequent complication in COVID-19, its consequences remain unknown. We performed pulmonary function tests, echocardiography and computed tomography pulmonary angiography and identified blood biomarkers in a cohort of consecutive hospitalized COVID-19 patients with pneumonia to describe and compare medium-term outcomes according to the presence of PE, as well as to explore their potential predictors. A total of 141 patients (56 with PE) were followed up during a median of 6 months. Post-COVID-19 radiological lung abnormalities (PCRLA) and impaired diffusing capacity for carbon monoxide (DLCOc) were found in 55.2% and 67.6% cases, respectively. A total of 7.3% had PE, and 6.7% presented an intermediate–high probability of pulmonary hypertension. No significant difference was found between PE and non-PE patients. Univariate analysis showed that age > 65, some clinical severity factors, surfactant protein-D, baseline C-reactive protein, and both peak red cell distribution width and Interleukin (IL)-10 were associated with DLCOc < 80%. A score for PCRLA prediction including age > 65, minimum lymphocyte count, and IL-1β concentration on admission was constructed with excellent overall performance. In conclusion, reduced DLCOc and PCRLA were common in COVID-19 patients after hospital discharge, but PE did not increase the risk. A PCRLA predictive score was developed, which needs further validation.

## 1. Introduction

More than 700 million individuals have been infected with the SARS-CoV-2 virus worldwide [1]. Coronavirus disease 2019 (COVID-19) is a heterogeneous disorder with a mortality rate of 1–3%. Most patients fully recover from the acute infection, but given the large number of individuals being infected, the long-term consequences are areas of concern, with the pulmonary and the cardiovascular systems being the target organs that are commonly affected [2].

Post-COVID-19 radiological lung abnormalities (PCRLA) are quite frequent in the follow-up of patients after COVID-19 pneumonia [3]. Most typical radiological sequalae are ground glass opacities with peripheral and diffuse distribution, with prevalence rates ranging from 7 to 92% [4,5,6,7,8,9,10,11,12]. Pulmonary function alterations (mainly a decrease in diffusing capacity for carbon monoxide corrected for hemoglobin (DLCOc)) have also been found after COVID-19, and they have been associated with the severity of radiological abnormalities. This indicates a potential interstitial lung disease (ILD) as a comorbidity of COVID-19, but with such a wide range of prevalence, it is difficult to reach clear conclusions [5,6,8,9,10,13,14]; however, these pulmonary abnormalities seem to improve over time [15].

Data of cardiac consequences of COVID-19 are limited. A recent case series has shown abnormalities in the magnetic resonance of patients who reported cardiac symptoms after being discharged in up to 50% of recently recovered patients. The right ventricle (RV) seems more likely to be affected compared to the left ventricle [16,17,18], and pulmonary hypertension (PH) is a potentially fatal consequence that has been considered and needs to be addressed. Pagnesi et al. [19] found a 12% of PH prevalence during hospitalization among mild–moderate COVID-19 patients, which was associated with worse prognosis. Estimated pulmonary artery systolic pressure by echocardiography after recovery was higher, according to the severity of pneumonia, and echocardiography markers of subclinical RV systolic dysfunction were impaired compared to patients without pneumonia [18]. However, other studies have described high prevalence of cardiac involvement independent of COVID-19 severity [16,19,20,21]. Few studies have evaluated PH after COVID-19, with prevalence rates ranging from 0 to10% [11,20,21,22].

On the other hand, pulmonary embolism (PE) has been described as a very frequent vascular complication of COVID-19 [23,24]. Both PE and microangiopathy could increase the incidence of PH [25]. Furthermore, a high risk of ILD has been found following PE in non-COVID-19 patients. Therefore, it could be speculated that PE and ILD could share some pathogenetic pathways, or even that a causality connection may exist in which PE activates and promotes local inflammation and fibrosis cascade in the lung parenchyma [26]. Evidence of long-term consequences of patients with PE and COVID-19 is still very scant and heterogeneous, showing a PH incidence ranging from 0 to 50% [27,28,29]. Only one small retrospective study evaluated pulmonary function at three months after COVID-19 in patients with PE, showing more dyspnea and a slightly higher impairment of pulmonary function compared with those without PE [30], but no differences in the rate of ILD. All of them were limited mainly due to the inclusion of patients who underwent computed tomography pulmonary angiography (CTPA) only when PE was suspected, and in whom follow-up was not systematically performed. 

While most of these studies suggest that a substantial number of COVID-19 survivors presented cardio-pulmonary sequelae, the fact that a substantial variation was observed stands out, which could be explained by samples with considerable variability in COVID-19 severity, heterogeneous inclusion criteria, different follow-up time, PH definition, treatment during follow-up, as well as certain underlying medical comorbidities [11,19,20,21,22,31]. Furthermore, alveolar damage, endothelial injury, hypoxemic vasoconstriction, systemic and localized immune responses, direct viral effects, and host response have been postulated as potential mechanisms, but data are still scant, and they are probably multifactorial [32]. Additionally, a global and systematic evaluation with lung function, exercise test, CTPA, blood biomarkers, and echocardiography in all patients was never completed.

Accordingly, the main aim of this study was to describe and compare medium-term respiratory and cardiovascular outcomes according to the presence of PE in a cohort of patients with COVID-19, in whom PE was systematically screened. As secondary objectives, (1) we compared baseline blood biomarkers according to the presence of respiratory and cardiovascular consequences; (2) we assessed key factors associated with abnormalities in DLCO and in CTPA; and (3) we developed a prognostic model to predict PCRLA in this cohort of patients with COVID-19.

## 2. Results

### 2.1. COVID-19 Pneumonia Population during Hospitalization

**Figure 1:**
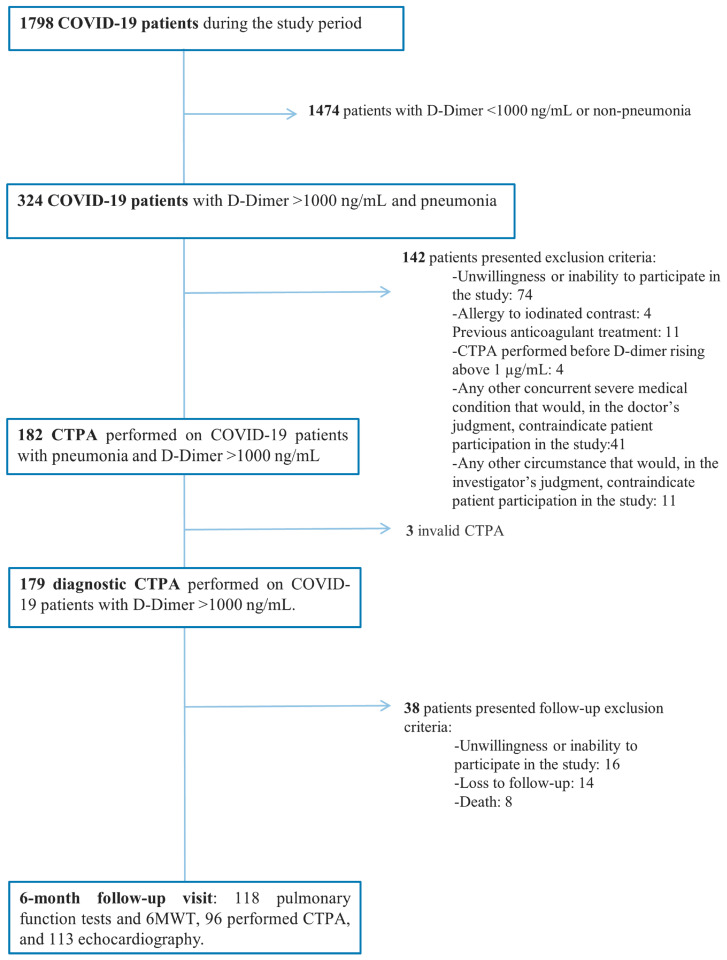
Flow Chart.

A total of 141 patients were included in the follow-up, and 56 of them had PE during hospitalization (Figure 1).

Table 1 describes anthropometric and clinical characteristics of hospitalized patients. The median age was 62 (54–72) years. No significant differences were found in age, BMI, smoking, clinical characteristics, and physical examination. Although the rate of patients requiring ICU was similar, patients without PE required the prone positioning in a higher proportion. No differences were found in the number of subjects requiring invasive/non-invasive ventilatory support or oxygen by high flow nasal cannula. Moreover, pharmacological therapy was comparable during hospitalization (Table 1). 

### 2.2. Clinical Characteristics at Follow-up 

Dyspnea was the most common symptom at the follow-up visit (36.8%), followed by asthenia (24.3%), arthralgias (15.9%) and headache (10.3%). No differences between PE and non-PE patients were found in clinical symptoms at the follow-up visit (Supplementary Table S1).

Additionally, dyspnea was more frequent in the subgroup of patients with DLCOc < 80% (42.6% vs. 21.9%, *p* = 0.047). No difference was found in the remaining clinical symptoms according to the presence of DLCOc < 80% or abnormal CTPA. 

### 2.3. Respiratory and Cardiovascular Consequences 

#### 2.3.1. CTPA Findings at Follow-up

**Figure 2:**
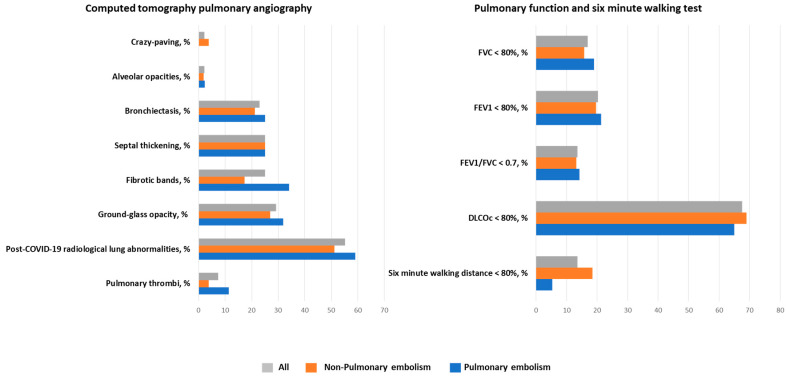
Radiological and pulmonary function findings at 6-month follow-up. Abbreviations: DLCOc, diffusing capacity for carbon monoxide corrected for hemoglobin; FEV1, forced expiratory volume in the first second; FVC, forced vital capacity.

A total of 96 patients underwent a CTPA at follow-up. The median time from CTPA performed during hospitalization to the follow-up CTPA was 182 (163–205) days, with no differences between groups. Figure 2 shows the CTPA findings in all patients, and according to outcome (with and without PE). We found PE in seven patients (7.3%) in the follow-up CTPA, and two of them were in the non-PE group. The remaining five patients had their thrombi at the same location as the previous episode, which represents a 11.4% of residual thrombosis at medium-term follow-up. Moreover, these seven patients were similarly distributed depending on whether they had PCRLA or DLCOc < 80%.

PCRLA at follow-up was found in 55.2% of the patients. The main findings were ground-glass opacities (29.2%), fibrotic band and septal thickening (25%), and bronchiectasis (22.9%). Other findings were alveolar opacities, crazy-paving, and pleural effusion in less than 5% of cases. There were no significant differences in the follow-up CTPA findings between patients with and without previous PE. Fibrotic bands were found in a higher proportion in PE compared with non-PE patients, although this difference failed to reach statistical significance (17.3% vs. 34.1%, respectively; *p* = 0.058) (Figure 2 and Supplementary Table S2).

#### 2.3.2. Pulmonary Function and Six-Minute Walking Test

Pulmonary function and six-minute walking tests were performed in 118 patients at follow-up. The median time from CTPA performed during hospitalization to the follow-up pulmonary function and six-minute walking test was slightly longer among those patients in the PE group: 188 (169–214) vs. 177 (155–198) days. On average, median FVC and FEV_1_ were within normal range at follow-up (92% [83–101%] and 94% [85–105%], respectively). Median DLCOc was 72% (58–87%), and 75 patients (67.6%) had impaired diffusion capacity (DLCOc < 80% predicted). Mean distance in 6MWT was 510 meters (437–570), and only 13.6% of patients had a distance walked below 80%.

No significant difference was found either in spirometry, DLCOc and 6MWT values, or in the percentage of patients with impaired lung or 6MWT tests between the PE and non-PE groups (Table 2 and Figure 2).

A total of 79 patients had both CTPA and pulmonary function data. No differences were identified in the percentage of patients with DLCOc values below 80% depending on the presence of PCRLA (67.6% vs. 75.6%).

#### 2.3.3. Echocardiography Findings

A total of 113 patients were evaluated with an echocardiography at follow-up. The median time from CTPA performed during hospitalization to the follow-up echocardiography was 171 (150–189) days. Mean left ventricular ejection fraction (LVEF) was 63.78 ± 6.54%. Only three patients had mild or moderate left ventricular systolic dysfunction. Mean tricuspid annular plane systolic excursion (TAPSE) was 22.34 ± 3.17 mm. Overall, the medians of tricuspid regurgitation pressure gradient and pulmonary artery pressure systolic were 22 [19,20,21,22,23] and 27 [23,24,25,26,27,28] mmHg, respectively. We did not find significant differences in any of these echocardiography characteristics among the groups (Supplementary Table S3).

There were three patients (6.7%) with intermediate–high probability of pulmonary hypertension at follow-up among those with previous PE, and only one (1.5%) in the non-PE group, although this did not reach statistical significance.

### 2.4. Laboratory Findings

#### 2.4.1. Baseline Blood Test on Admission

The descriptive analysis of both groups for the baseline laboratory findings are shown in Table 3. We found a higher baseline, in both leucocyte and neutrophil counts, in PE patients than in the non-PE group. Furthermore, cholesterol concentration on admission was slightly lower in non-PE patients. The remaining parameters of baseline blood count, biochemical profile, coagulation function, and arterial blood test did not differ between the two groups.

#### 2.4.2. Inflammatory and Thrombotic Biomarkers on Admission

Interleukin-10 (IL-10) peak values were lower in PE patients compared with non-PE patients. We also found significant differences in PDW and NRL baseline levels among both groups. In addition, higher baseline and peak D-Dimer values were found in patients with PE when compared to patients without PE.

We next analyzed the former biomarkers according to the DLCOc values. Red blood cell distribution width (RDW), IL-10 and N-terminal pro hormone B-type natriuretic peptide (NT-pro-BNP) peak values were higher in patients with DLCOc < 80% at follow-up. Furthermore, baseline platelet count was lower among these patients. However, when evaluating these laboratory data between patients with or without PCRLA during follow-up, significant differences were only found in D-dimer peak values during admission (3364 (2329–7281) vs. 72,410 (1616–4032) ng/mL, respectively) (Table 4).

#### 2.4.3. Blood Test at Follow-up

Blood tests were performed on all patients during their follow-up visit. D-dimer levels were significant higher in non-PE patients than in PE patients (134 (72–216) vs. 84.5 (50–156) ng/mL, respectively), although no significant differences were found in the proportion of patients with elevated D-dimer values, in blood counts, or NT-pro-BNP, lactate dehydrogenase (LDH), ferritin, C-reactive protein (CRP), and erythrocyte sedimentation rate (ERS) levels at follow-up (Table 3).

#### 2.4.4. Biomarkers of Interstitial Lung Disease, Inflammation, and Coagulation Collected on Admission

The analyses of interstitial lung disease, inflammatory and thrombotic biomarkers on admission are shown in Table 5. S1PR1 levels were lower in those patients with impaired diffusion capacity (1.30 [0.80–3.62] vs. 4.15 [1.28–5.04] ng/mL, respectively). Additionally, the SP-D concentration was higher in patients with DLCOc < 80% (8.49 [2.94–25.49] vs. 3.34 [0.52–8.06] ng/mL, respectively).

Table 5 shows that patients with PCRLA after hospital discharge had significantly lower concentrations of plasminogen and significant higher IL-1-β concentration during hospital admission. Moreover, macrophage inflammatory protein 4-α, VE-Cadherin, and TNF-α levels showed different values, but these differences did not reach statistical significance. No other statistical differences were found between both groups in the remaining biomarkers analyzed in the study when evaluating the existence of DLCO impairment or PCRLA.

### 2.5. Factors Associated with Lung Abnormalities

Supplementary Table S4 presents the results of univariate analysis of risk of abnormal DLCO at medium-term follow-up. Age > 65 years, WHO severity classification, CURB-65, high flow oxygen, maximum FiO_2_, SP-D, baseline CRP, both peak red cell distribution width and IL-10, as well as length of hospital, ICU and oral tracheal intubation stay were associated with impaired DLCOc. 

A total of 10 variables related to the presence of PCRLA in the regression analysis were dichotomized to construct a PCRLA predictive score (Supplementary Table S5). After dichotomization, multivariable logistic regression analysis led to a selection of three variables (Supplementary Table S6). Table 6 shows the constructed prediction score with the calculated weight and cut-off points of the variables. The score included age >65 years, minimum lymphocyte count, and IL-1β concentration. 

The AUC-ROC of the score was 0.81 (95% CI: 0.72–0.91), *p* < 0.001 (Supplementary Figure S1). The suggested score ranged from 0 to 6 points. The prevalence of PCRLA was low (3.8%) at 0–1 point, moderate (13.2%) at 2 points, high (37.7%) at 3–4 points, and very high (45.3%) at 5–6 points. A value ≥ 3 was predictive of PCRLA with a sensitivity of 83%, a specificity of 65.1%, and a false positive rate of 34.9%, but improved if the score ≥ 4 and ≥ 5 to a specificity of 80.6% and 82.8%, and false positive rate of 16.3% and 11.6%, respectively. Supplementary Table S7 summarizes sensitivity, specificity, and positive and negative predictive values of different cut-off points.

Missing data values for all variables are collected in Supplementary Tables S8–S13.

## 3. Discussion

This is the largest prospective study to date that systematically explores clinical characteristics, pulmonary function, CTPA, and echocardiography findings at medium-term follow-up after COVID-19 with pneumonia hospitalization according to the presence of PE. In addition, we also analyzed several laboratory variables, including baseline, peak, and follow-up blood tests values, as well as biomarkers of interstitial lung diseases, inflammation, and thrombosis during hospitalization. Finally, we developed a promising predictive score of PCRLA at medium-term, taking into account several laboratory and clinical variables on admission.

### 3.1. Clinical Characteristics, Respiratory and Cardiovascular Consequences at Medium-Term Follow-up

Even though the COVID-19 pandemic started four years ago, there are new findings from follow-up studies already showing that impaired diffusion capacity and PCRLA are quite prevalent after SARS-CoV-2 infection. Ground glass opacities with peripheral and diffuse distribution are the most common of the latter, but variable rates of parenchymal bands, reticulation, septal thickening, and bronchiectasis have also been described. Nevertheless, the prevalence of COVID-19 lung sequelae is highly heterogeneous among studies, and its determinants remain unknown [3,4,5,6,7,8,9,10,11,12,13,14,15]. Our study revealed comparable findings, with ground glass opacifications and DLCOc < 80% being the most common lung sequalae at medium-term, and with 44.8% of patients having complete resolution of lung infiltrates. Overall, our data showed no differences in clinical characteristics, pulmonary function, echocardiography and CTPA findings at medium-term follow-up between patients with and without PE during admission. As far as we know, there is only one previous study comparing pulmonary function in patients with and without PE during COVID-19 [30]. They included 68 patients (24 had previous PE) and found that patients with PE had lower FVC and DLCO when compared with non-PE patients at three-month follow-up [30]. However, similar to us, they found a high proportion of patients with pulmonary diffusion impairment. Half of the patients with PE had a DLCO < 80%, although without significant differences between groups; moreover, they had more dyspnea and worse oxyhemoglobin saturation during 6MWT. These contradictory data in pulmonary function could be related to a smaller sample size, with significant differences in clinical characteristics of patients, since most of them had had mild COVID-19, as well as to a distinct follow-up time, which has been shown to be quite determinant, as an improvement in FVC is usually observed over time after discharge [9,33]. Finally, the inclusion of patients with a clinical suspicion of PE, and not all consecutive patients, as it is the case in our study, may also influence the prevalence of cardiorespiratory consequences at the follow-up. Nevertheless, our study maintains some concordances with this previous study regarding the absence of differences in radiological findings during follow-up between patients with and without PE [30], which seems also to improve over time, as they found PCRLA in 78.9% of patients at three months. Another small study also reported PCRLA in 46.7% of PE patients at six months [34], which is similar to the 59% of patients with PCRLA at medium-term follow-up of the present study. Lastly, it is interesting to highlight that we observed that the presence of fibrotic bands was twice as high in patients with PE (34.1% vs. 17.3%, respectively; *p* = 0.058). Although this difference did not reach statistical significance, it would be of interest to explore this question in further studies with larger samples.

Given the elevated occurrence of PE in COVID-19 and its potentially distinct underlying mechanisms [25,35], thrombus resolution could be less pronounced, contributing, among other factors, to a higher risk of pulmonary hypertension. However, residual thrombosis was found in 5 of the 44 patients who were followed up with CTPA (11.4%) in the present study, which do not seem to be different from non-COVID-19 patients, in whom the persistence of thrombi at six months was 16% [36]. Resolving pulmonary clots after COVID-19 have been studied in few previous studies, yet with a high variability of reported rates ranging from 4.4 to 57% by method for identifying residual thrombi, thrombi location, and certain underlying medical comorbidities, as well as both time from the PE episode and duration of anticoagulation treatment [29,30,34,37].

Moreover, in our study, both baseline and peak D-dimer values were higher in patients with PE during hospitalization, and at medium-term follow-up, D-dimer values were much lower across all patients. Median follow-up D-dimer concentrations were within the normal range, although patients with previous PE had lower concentrations, most likely due to anticoagulant treatment given, which is known to significantly reduce D-dimer levels in non-COVID-19 PE patients. Thus, a large study showed only 5.1% of general PE patients remain above the D-dimer cut-off point while on anticoagulants [38]. Furthermore, D-dimer levels decrease over time but remain elevated in 15–42% of post-COVID-19 patients, with variations based on disease severity, follow-up duration, and treatments (mainly anticoagulants) [11,39,40,41]. However, two important issues must be considered. First, D-dimer reference values should be adjusted for age in patients over 50, but not all the studies did so, leading to discrepancies (in our study, 19.2% vs. 6.7% had high D-dimer when age-adjusted). Second, inconsistencies in reporting D-dimer units (DDU vs. fibrinogen equivalent units (FEU)) can lead to misinterpretations, as FEU values are twice those of DDU, and in some studies, the types of units used have not been specified [11,40,41].

### 3.2. Laboratory Findings and Inflammatory and Thrombotic Biomarkers on Admission 

SP-D is a surfactant protein secreted by pulmonary alveolar type II epithelial cells that is crucial for the lung’s innate immune response and is elevated when the alveolo-capillary barrier is impaired [42]. Increased SP-D levels have been found in severe COVID-19 patients [43] and in those patients with DLCO impairment six months post-discharge [14]. We found higher SP-D levels during hospitalization in patients with later DLCOc < 80% or PCRLA at medium-term, although the latter difference was not significant (*p* = 0.08). This suggests SP-D may indicate not only acute lung damage but also medium-term respiratory complications.

S1PR1 signaling has also been involved in activation in the immune system as well as in regulating endothelium function and vascular permeability [44,45]. S1PR1 has been implicated in the modulation of wound healing following injury and significantly contributes to the onset of ARDS as well as the potential progression to pulmonary fibrosis [45]. Interestingly, we found lower S1PR1 levels in those patients with DLCO dysfunction. Very recent data suggest that the overexpression of S1PR1 signaling reduces post-viral pulmonary fibrosis, which supports the research of S1PR1 agonists as a potential treatment for COVID-19 respiratory consequences [46,47]. 

IL-10 increased concentrations have been correlated both with COVID-19 severity and DLCO measured six months after hospitalization [48,49]. Similarly, our data revealed higher peak IL-10 levels during hospitalization in those patients with DLCOc < 80% at medium-term. In this regard, it is interesting to underline that IL-10 promotes the viability and collagen synthesis of primary pulmonary fibroblasts in pulmonary fibrosis [50].

RDW, D-dimer, plasminogen and platelet count are routine laboratory tests. Higher RDW and D-dimer values as well as lower plasminogen and platelet count have been associated with increased mortality risk during COVID-19 hospitalization [51,52,53,54,55]. Patients with idiopathic pulmonary fibrosis with elevated levels of RDW present more advanced disease and lower DLCO, [56]. In line with these concepts, we found more elevated peak RDW during hospitalization in patients with DLCOc < 80% at follow-up. As mentioned above, baseline platelet count was lower when patients had a reduced DLCOc [14,57]. Finally, and according with our results, peak D-dimer value during hospitalization was a predictive variable of fibrotic-like changes at follow-up [58,59]. 

### 3.3. PCRLA Predictive Score

Age > 65, minimum lymphocyte count, and IL-1β concentration during hospitalization were the significant included variables in the PCRLA risk score. The relationship between COVID-19 and pulmonary fibrosis is highly intricate, with numerous factors and confounding variables that still need clarification. It has been reported that, among others, IL-1β, the degree of lymphopenia, and advanced age on admission may already not only affect the prognosis [60,61], but also the risk of developing long-term respiratory impairment [62,63,64,65,66]. Intriguingly, IL-1β stimulates fibroblasts to produce both collagen and fibrin, and an animal model has demonstrated that acute lung injury could lead to progressive fibrotic changes that are mediated by IL-1β [67]. However, the current understanding does not clarify which individuals can recover from SARS-CoV-2-induced lung injury, while some persist in developing epithelial dysfunction and abnormal repair mechanisms that could result in fibrosis. Additionally, at present, there are no therapies for preventing or treating PCRLA. Our PCRLA predictive score showed excellent accuracy (AUC-ROC of 0.81; 95% CI: 0.72–0.91), and 80.6% of patients were correctly identified as having PCRLA at medium-term when values were greater than 2. Interestingly, when the PCRLA predictive score was ≤1, the prevalence of PCRLA was low (3.8%). However, despite the promise shown by this score, the flaws of this score must be acknowledged: it has different accuracy values (sensitivity, specificity, PPV, NPV) depending on the chosen cutoff point (Supplementary Table S7), and when the score was at least 3 points, the specificity was 65.1% and the false positive rate 34.9%, leading to an increase in the cost of diagnostic testing, anxiety burden for the patient, and radiation risk. Moreover, excessive referral may overburden diagnostic services, to the detriment of other activities. Therefore, it is essential to conduct internal and external validations, as well as cost-effectiveness studies before implementing it in clinical settings.

### 3.4. Strengths and Limitations

The present study has several strengths, such as its prospective enrollment, clear inclusion/exclusion criteria, and intensive characterization from the point of view of clinical, imaging, and complete laboratory tests, including inflammatory and coagulation biomarkers, which were measured during admission. Additionally, this is the largest prospective study exploring clinical characteristics, pulmonary function, and laboratory, CTPA and echocardiography findings at medium-term follow-up between patients with and without PE during primary infection, which had been systematically explored during hospitalization regardless of symptoms. Furthermore, this study represents the whole spectrum of hospitalized COVID-19 patients (ICU and non-ICU) during follow-up. However, there are some potential limitations to consider. First, this study was carried out before the Omicron variant had become the dominant form of SARS-Co-2 and with low vaccination rates (none of the patients were vaccinated against SARS-CoV-2), all of which may have had an impact on the development of cardiopulmonary complications, as well as on the biomarkers concentration. Second, this study lacks a control group with patients who did not have COVID-19. Third, due to the relatively small sample size, the single-center management, and the medium-term follow-up, the results need to be interpreted carefully, although the sample was sufficiently comprehensive to demonstrate some significant differences. In addition, most previous studies evaluating follow-up sequelae included smaller sample size [13,18,30,68]. Finally, CPTA was routinely performed in patients with D-dimer > 1000 ng/mL with no exclusion criteria; therefore, the usefulness of the CPTA score and the results of our study among the remaining patients with SARS-CoV-2 pneumonia are unknown.

## 4. Material and Methods

### 4.1. Study Design

This is a cohort follow-up study of a previous prospective study performed to determine the prevalence of PE in patients admitted because of COVID-19 pneumonia. A detailed description of the study aims and protocol, including inclusion and exclusion criteria, is outlined elsewhere [23]. Briefly, all consecutive hospitalized patients with confirmed COVID-19 pneumonia and D-dimer levels >1000 ng/mL (DDU) during hospitalization underwent a CTPA, which classified them by the presence of PE. Patients lacking follow-up visit were excluded from the analyses. Additionally, patients were excluded from PCRLA analysis and pulmonary function examinations if they had previous ILD or severe mental disability. COVID-19 was considered upon a positive result on polymerase chain reaction (PCR) testing of a nasopharyngeal sample. Strengthening of the reporting of observational studies in epidemiology (STROBE) statement was followed [69].

### 4.2. Description of Investigations Undertaken

Patients with PE during hospitalization were on therapeutic doses of anticoagulant therapy for at least six months, and they were scheduled for a medium-term follow up visit. All the included patients were contacted by a nurse through a phone call and invited to participate in the follow-up study at least 8 weeks after the first CTPA. The patients were contacted in the same order as the date when the first CTPA was performed to schedule the following clinical visits and tests. If the follow-up appointment was missed, the patient was given two opportunities to reschedule the visit.

Demographic, clinical, and laboratory examinations were collected from all subjects both at the time of admission and at follow-up. All patients underwent pulmonary function tests, including spirometry, DLCOc, six-minute walking test (6MWT), and echocardiography, at follow-up. The following spirometry parameters were measured: forced expiratory volume in the first second (FEV_1_), forced vital capacity (FVC), FEV_1_/FVC ratio, DLCOc, and transfer coefficient of the lung for carbon monoxide (KCO) were measured by the single-breath DLCO test. All parameters were expressed as percentages of the predicted value (%) and considered reduced if below 80%, in accordance with the European reference values [70,71,72,73]. More details are shown in the Supplementary Materials. Moreover, a second CTPA was also carried out, and the following findings were defined in the Fleischner Society glossary [74] by two experienced radiologists: (a) ground grass opacifications, (b) fibrotic bands, (c) septal thickening, (d) bronchiectasis, (e) crazy-paving, and (f) alveolar pattern. PCRLA was defined when at least one of them was detected. 

### 4.3. Biomarkers 

Blood was collected in EDTA tubes during hospitalization when first CTPA was performed and was then centrifuged at 600× *g* 10 min 4 °C to obtain the plasma, which was stored at −80 °C until analysis. Plasma concentrations of tumor necrosis factor (TNF)-α receptor-1, TNF-α receptor-2, TNF-α, interleukin (IL)-1-β, carbohydrate antigen 15-3 (CA 15-3), matrix metalloproteinase 7, P selectin, macrophage inflammatory protein 4-α, galectin, and epidermal growth factor receptor in serum were analyzed using a human cytokine magnetic bead panel (Merck Millipore, Billerica, MA, USA), in accordance with the manufacturer’s instructions. Plasminogen (Merck Millipore, Billerica, MA, USA), protein C, VE-Cadherin (Abcam, Cambridge, UK), sphingosine 1 phosphate receptor-1 (S1PR1) and surfactant protein D (SP-D) (Labclinics, Barcelona, Spain) were quantified in serum by ELISA, following the manufacturer’s instructions. Assay sensitivity according to the manufacturer was: 8 pg/mL for TNF-RII, 12 pg/mL for TNF-RI, 0.16 pg/mL for TNF-α, 0.03 pg/mL for CA15-3, 97 pg/mL for matrix metalloproteinase 7, 3.3 pg/mL for macrophage inflammatory protein 4-α, 42 pg/mL for epidermal growth factor receptor, 0.14 ng/mL for plasminogen, 66 ng/mL for protein C, 331 pg/mL for VE-Cadherin, 0.094 ng/mL for S1PR1 and 0.938 ng/mL for SP-D, 0.051 ng/mL for P selectin, and 0.002 ng/mL for galectin.

### 4.4. Ethics Statement

The Institutional Ethics Committee of the Balearic Islands approved the study (IB 4197/20 PI), and all subjects gave their written informed consent. Only patients with a critical clinical condition gave verbal consent instead, in front of at least two witnesses.

### 4.5. Statistical Analysis

Descriptive statistics included frequencies and percentages for categorical variables and medians and interquartile ranges (IQRs) for continuous variables. Comparisons were determined by the Mann–Whitney *U*-test for continuous variables, and by the χ^2^-test or Fisher’s exact test for categorical variables. 

The total population of the study was divided into two groups: patients with PE and patients without PE. Furthermore, the total population of the study was divided into four additional groups to assess key factors associated with abnormalities in DLCO and in CTPA: patients with DLCOc < 80% vs. DLCOc ≥ 80%; and patients with PCRLA vs. non-PCRLA. Also, odds ratio (OR) 95% confidence intervals (CI) were calculated for each outcome variable. Finally, baseline and peak variables related to the presence of PCRLA in the regression analysis were dichotomized to construct predictive score. Youden’s index criteria were used to determine the cut-off point for each variable. After dichotomization, these variables were included in a logistic regression model. Relative weight analyses were used to calculate the relative weight of each variable. The sum of the relative weight of all the variables included in the score corresponded to its total value. The discriminatory capacity of the score was evaluated by a receiver operating characteristic (ROC) curve analysis, and the sensitivity, specificity, and positive and negative predictive values of different cut-off points were calculated. The missing data were considered at random. No imputations were made, and pairwise deletion was performed. Percentages were calculated by category after exclusion of patients with missing values for that variable. Differences were considered statistically significant at a 2-tailed *p*-value of< 0.05. The statistical software used was SPSS v.26 (IBM Corporation, Armonk, NY, USA).

## 5. Conclusions

In conclusion, impaired DLCOc and PCRLA are considerably common after SARS-CoV-2 pneumonia at medium-term follow-up, and those patients who developed PE during admission were not at increased risk. Laboratory and clinical data and some specific biomarkers such as platelet and lymphocyte counts, RDW, D-dimer, SP-D, S1PR1, IL-1β, IL-10, NT-proBNP, and plasminogen are significantly different in patients with medium-term respiratory complications. Finally, a three-variable PCRLA predictive score was developed, which needs further validation.

There is still much to understand and be concerned about regarding the long-term impacts of COVID-19, along with other associated health conditions. Our study provides valuable information that may help in understanding its molecular mechanisms, pathogenesis, and diagnosis, as well as in identifying therapeutic targets in the treatment of PCRLA and pulmonary fibrosis in patients with COVID-19. Nevertheless, further and larger multicenter studies including non-hospitalized patients are clearly needed to better clarify the cardiovascular and respiratory consequences of COVID-19 hospitalized patients.

## Figures and Tables

**Table 1:** Baseline anthropometric and clinical characteristics of patients admitted because of COVID-19 pneumonia.

Blanco	All (n = 141)	Non-PE (n = 85)	PE (n = 56)	*p* Value
Age, yrs.	62 (54–72)	61 (54–73)	63 (54–70)	0.99
Age >65 yrs.	63 (44.7)	37 (43.5)	26 (46.4)	0.74
BMI, kg/m^2^	28.9 (26.2–31.6)	28.5 (26–31.2)	29.8 (26.6–31.6)	0.13
Smoking status				0.84
Current smoker, n (%)	6 (4.3)	3 (3.5)	3 (5.4)	
Former smoker, n (%)	44 (31.2)	26 (30.6)	18 (32.1)	
Smoking, Pack-year	0 (0–10)	0 (0–10)	0 (0–10)	0.73
Hypertension, n (%)	65 (46.1)	40 (47.1)	25 (44.6)	0.78
Diabetes mellitus, n (%)	39 (27.7)	23 (27.1)	16 (28.6)	0.84
Cardiovascular disease, n (%)	25 (17.7)	11 (12.9)	14 (25)	0.07
Cerebrovascular disease, n (%)	9 (6.4)	5 (5.9)	4 (7.1)	0.76
Chronic kidney disease, n (%)	11 (7.8)	5 (5.9)	6 (10.7)	0.29
COPD, n (%)	5 (3.5)	2 (2.4)	3 (5.4)	0.34
Asthma, n (%)	7 (5)	4 (4.7)	3 (5.4)	0.86
COVID-19 Admission				
Physical examination				
Respiratory rate, breaths per min	24 (19–28)	24 (19–28)	22 (19–28)	0.59
Heart rate, beats per min	86 (74–99)	87 (76–99)	85 (71–103)	0.57
Systolic BP, mm Hg	125.00 (114–138)	125 (114–139)	125 (116–135)	0.92
Diastolic BP, mm Hg	71 (64–80)	70 (63–78)	72 (64–81)	0.52
Temperature, °C	36.9 (36.1–37.6)	36.9 (36.2–37.6)	36.9 (36.1–37.6)	0.88
CURB 65	0.64	1 (0–2)	1 (1–2)	0.36
WHO severity classification				
WHO category 3	6 (4.3)	6 (7.1)	0 (0)	0.19
WHO category 4	67 (47.5)	41 (48.2)	26 (46.4)	
WHO category 5	20 (14.2)	9 (10.6)	11 (19.6)	
WHO category 6	27 (19.1)	17 (20)	10 (17.9)	
WHO category 7	21 (14.9)	12 (14.1)	9 (16.1)	
Treatment in hospital				
High flow oxygen, n (%)	37 (26.4)	21 (25)	16 (28.6)	0.64
Non-Invasive ventilation, n (%)	3 (2.1)	1 (1.2)	2 (3.6)	0.33
Invasive ventilation, n (%)	48 (34.3)	29 (34.5)	19 (33.9)	0.94
Prone position, n (%)	19 (13.5)	16 (18.8)	3 (5.4)	0.02
Azithromycin, n (%)	24 (17)	15 (17.6)	9 (16.1)	0.78
Hydroxychloroquine, n (%)	32 (22.7)	15 (17.6)	17 (30.4)	0.77
Lopinavir/ritonavir, n (%)	25 (17.7)	12 (14.1)	13 (23.2)	0.24
Remdesivir, n (%)	21 (15)	13 (15.5)	8 (14.3)	0.85
IFNb, n (%)	2 (1.4)	1 (1.8)	1 (1.2)	0.76
Tocilizumab, n (%)	35 (24.8)	15 (26.8)	20 (23.5)	0.66
Systemic Corticosteroids, n (%)	121 (85.8)	73 (85.9)	48 (85.7)	0.97
ICU, n (%)	59 (41.8)	33 (38.8)	26 (46.6)	0.37

Abbreviations: BMI, body mass index; BP, blood pressure; COPD, chronic obstructive pulmonary disease; ICU, intensive care unit; IFNb, interferon beta; PE, pulmonary embolism; WHO (world health organization) severity. 3: hospitalized, no oxygen therapy; 4: hospitalized, oxygen mask or nasal prongs; 5: hospitalized, noninvasive mechanical ventilation (NIMV) or high-flow nasal cannula (HFNC); 6: hospitalized, intubation and invasive mechanical ventilation (IMV); 7: hospitalized, IMV + additional support such as pressors or extracardiac membranous oxygenation (ECMO). As this was a hospital-based study, the patients included corresponded to categories 3 to 7 of the WHO ordinal scale.

**Table 2:** Pulmonary function and six-minute walking test at medium-term follow-up.

Columna1	All (n = 119)	Non-PE (n = 76)	PE (n = 43)	*p* Value
FVC, (%)	92 (83–101)	92 (83–101)	91 (83–102)	0.87
FEV1, (%)	94 (85–105)	94 (85–105)	95 (84–101)	0.80
FEV1/FVC, (%)	78.5 (41.6–83.1)	78.9 (75–83.3)	76.9 (74–82)	0.32
DLCOc, (%)	72 (58–87)	71 (58–88)	73 (59–88)	0.42
KCOc, (%)	77 (66–87)	4.1 (3.2–4.6)	3.9 (3.4–4.5)	0.56
Walking distance, (m)	510 (437–570)	503 (428–540)	512 (4663–584)	0.07
Walking distance, (%)	100 (89–114)	98 (88–113)	105 (96–114)	0.40
Resting oxygen saturation, (%)	97 (96–98)	97 (96–98)	97 (96–98)	0.78
End-exercise oxygen saturation, (%)	96 (94–97)	96 (94.5–97)	96 (94–97)	0.29
Lowest oxygen saturation, (%)	95 (93–95)	95 (93–95.5)	94 (93–95)	0.49

Abbreviations: DLCOc, diffusing capacity for carbon monoxide corrected for hemoglobin; FEV1, forced expiratory volume in the first second; FVC, forced vital capacity; PE, pulmonary embolism.

**Table 3:** Baseline and medium-term follow-up laboratory data.

	All (n = 141)	Non-PE (n = 85)	PE (n = 56)	*p* Value
Blood count, baseline				
Hemoglobin, g/dL	13.7 (12.6–15)	13.7 (12.2–15)	13.8 (12.9–15)	0.72
Leucocyte count, 10^3^/µL	13.8 (9–19.8)	14.6 (10–20.4)	11.1 (7.8–16)	0.04
Lymphocyte count, 10^3^/µL	1.1 (0.8–1.4)	1.1 (0.8–1.4)	1 (0.7–1.3)	0.37
Neutrophil counts, 10^3^/µL	6.1 (4.3–9)	5.7 (4–7.8)	7 (4.6–10)	0.03
Biochemical profile, baseline				
Glucose, mg/dL	124 (105–151)	120 (104–140)	130 (110–170)	0.16
ALT, U/L	31 (18–56)	27 (17–65)	36 (21–50)	0.85
Urea, mg/dL	35 (26–47)	32 (24–45)	38 (31–52)	0.08
Creatinine, mg/dL	0.8 (0.7–1.1)	0.8 (0.7–1)	0.9 (0.8–1.2)	0.08
Sodium, mEq/L	137 (135–140)	138 (135–140)	137 (135–139)	0.34
Potassium, mEq/L	4 (3.7–4.5)	4 (3.7–4.4)	4.1 (3.8–4.5)	0.23
Cholesterol, mg/dL	145 (121–170)	140 (121–160)	153 (122–187)	0.03
Triglyceride, mg/dL	135 (103–189)	126 (94–177)	152 (120–207)	0.05
Coagulation function, baseline				
PT, %	81 (71–89)	81 (73–90)	81 (67–87)	0.29
INR	1.13 (1.07–1.23)	1.13 (1.06–1.21)	1.13 (1.10–1.30)	0.13
Fibrinogen, mg/dL	690 (513–833)	686 (509–793)	724 (595–860)	0.18
Arterial blood test, baseline				
PaO_2_/FiO_2_ ratio	276 (217–324)	290 (225–323)	257 (214–324)	0.27
pH	7.46 (7.43–7.50)	7.46 (7.43–1.50)	7.46 (7.45–7.49)	0.50
PaO_2_, mmHg	63 (55–74)	62 (55–71)	64 (56–83)	0.18
PaCO_2_, mmHg	33 (29–36)	33 (30–36)	30 (28–35)	0.11
Follow-up laboratory findings				
Hemoglobin, g/dL	14 (12.8–15)	14.3 (12.9–15)	14 (12.7–14.8)	0.42
Leukocyte count, 10^3^/µL	6.6 (5.6–8.4)	6.7 (5.6–8.5)	6.3 (5.52–8.2)	0.53
Neutrophil count, 10^3^/µL	3.6 (2.7–4.7)	3.6 (2.7–4.8)	3.6 (2.9–4.6)	0.72
Lymphocyte count, 10^3^/µL	2.2 (1.8–2.8)	2.2 (1.9–2.7)	2.3 (1.6–2.8)	0.74
Platelet count, 10^3^/µL	232 (200–275)	230 (196–274)	245 (212–276)	0.34
ERS, mm/h	15 (7–30)	14 (7–23)	17 (10–36)	0.11
D-dimer, ng/mL	108 (60–203)	134 (72–216)	84 (50–141)	0.00
Elevated D-dimer, n (%)	8 (6.7)	7 (9.7)	1 (2.1)	0.13
CRP, mg/dL	0.27 (0.12–0.46)	0.27 (0.12–0.43)	0.26 (0.13–0.53)	0.61
Ferritin, ng/ml	70 (33–137)	81 (35–160)	57 (33–101)	0.07
LDH, U/L	200 (174–225)	195 (170–227)	209 (188–225)	0.21
NT-pro-BNP, pg/mL	70 (35–169)	60 (28–165)	86 (41–160)	0.29

Abbreviations: ALT, alanine transaminase; CPR, C reactive protein; FiO_2_, fraction of inspired oxygen; INR, international normalized ratio; LDH, lactate dehydrogenase; NT-pro-BNP, N-terminal pro hormone B-type natriuretic peptide; PaO_2_, arterial partial pressure of oxygen; PaCO_2_, arterial partial pressure of carbon dioxide; PE, pulmonary embolism; PT%, prothrombin time.

**Table 4:** Inflammatory and thrombotic biomarkers on admission according to outcome.

	Non-PE (n = 85)	PE (n = 56)	*p* Value	DLCOc ≥ 80% (n = 36)	DLCOc < 80% (n = 76)	*p* Value	No PCRLA (n = 43)	PCRLA (n = 53)	*p* Value
LDH									
Baseline, U/L	360 (291–531)	359 (287–493)	0.92	354.50 (293–464)	365 (291–528)	0.63	376.50 (288–494)	411 (299–588)	0.54
Peak, U/L	459 (343–626)	434 (345–586)	0.48	436 (349–523)	475 (355–616)	0.27	425 (334–600)	473 (349–615)	0.37
CRP									
Baseline, mg/dL	10.5 (4.8–19.2)	10.8 (4.6–19.2)	0.85	9.8 (5.0–19.0)	12.0 (5.3–23.3)	0.390	11.0 (5.2–18.4)	10.9 (4.8–22.7)	0.94
Peak, mg/dL	14.6 (7.7–21.8)	15.3 (8.5–25.8)	0.59	15.1 (7–21.7)	16.0 (8.8–26.8)	0.37	14.4 (6.9–19.3)	16.0 (7.7–26.8)	0.25
ESR									
Baseline, mm/h	69 (46–88)	68 (49–88)	0.77	65 (49–85)	69 (44.5–87.5)	0.88	74 (50–92)	66 (45–89)	0.29
Peak, mm/h	80 (57–104)	76 (65–94)	0.92	78.5 (62–103)	76 (60.5–99.5)	0.65	77 (62–101)	75.50 (60.50–99)	0.55
D-dimer									
Baseline, ng/mL	561 (237–1634)	1489 (321–5199)	0.01	534 (232–2307)	672 (314–2165)	0.46	918 (256–2228)	519 (294–2332)	0.74
Peak, ng/mL	2345 (1618–3583)	3653 (2774–8585)	0.00	2620 (1901–377)	2857 (1920–5230)	0.36	2410 (1616–4032)	3364 (2329–7281)	0.04
Ferritin									
Baseline, ng/mL	764 (360–1392)	612 (378–932)	0.18	811 (482–1433)	764 (391–1159)	0.57	582 (254–1017)	726 (471–1106)	0.18
Peak, ng/mL	1071 (551–2474)	865 (419–1689)	0.06	1057 (628–1760)	1068 (496–2290)	0.95	765 (348–2333)	1162 (527–2179)	0.16
Platelet count									
Baseline, 10^3^/µL	205 (173–295)	237 (175–312)	0.26	255 (189–312)	193 (156–289)	0.04	236 (182–312)	204 (151–277)	0.18
Peak, 10^3^/µL	409 (312–512)	363 (292–490)	0.12	401 (314–516)	373 (283–496)	0.12	387 (303–503)	373 (297–495)	0.78
Lymphocyte counts									
Baseline, %	14.6 (9.7–20.2)	11.0 (7.8–15.9)	0.36	16.0 (9.4–23.6)	12.50 (8.8–18.5)	0.15	15.60 (8.63–20.50)	11.70 (9.3–16.8)	0.18
Peak *, %	8.2 (4.7–13.4)	6.07 (4.8–8.9)	0.08	8.1 (5.3–15.2)	6.23 (4.5–10.1)	0.10	7.68 (5.06–14.15)	6.52 (4.8–9.8)	0.23
NLR									
Baseline	5.1 (3.5–8.4)	7.2 (4.8–10.6)	0.01	4.8 (2.9–8.8)	9.2 (3.9–9.5)	0.14	4.9 (3.4–9.7)	6.9 (4.5–0.1)	0.19
Peak	10.7 (5.9–18.6)	14.7 (9.8–18.9)	0.07	10.7 (4.8–17.1)	14.4 (8.0–20.8)	0.12	11 (5.8–18.1)	12.8 (8.0–19.3)	0.27
RDW, %									
Baseline	12.3 (11.9–13.0)	12.2 (11.9–12.9)	0.57	12.1 (11.9–12.8)	12.4 (12.0–13.2)	0.14	12.2 (12.0–12.9)	12.2 (11.8–12.7)	0.53
Peak	13.4 (12.5–16.3)	13.0 (12.5–14.3)	0.13	12.8 (12.2–13.4)	13.6 (12.7–15.1)	0.00	12.8 (12.4–13.4)	13.4 (12.6–14.9)	0.06
PDW, %									
Baseline	16.1 (15.7–16.7)	16.7 (16.2–17.2)	0.00	15.0 (15.8–16.9)	16.2 (15.8–16.8)	0.38	16.4 (15.9–16.8)	16.5 (16.0–16.9)	0.59
Peak	17.1 (16.8–17.7)	17.3 (17.0–18.0)	0.09	17.2 (16.8–17.7)	17.2 (16.9–17.7)	0.50	17.2 (16.9–17.7)	17.5 (17.0–17.8)	0.21
IL-6, pg/mL peak	66.0 (19.6–195.0)	59.4 (27.0–89.4)	0.59	59.0 (23.0–94.0)	56.0 (19.0–23.5)	0.45	42.0 (22.5–107.4)	65.0 (25–235.0)	0.24
IL-10, pg/mL peak	10.0 (6.7–17.1)	5.0 (3.3–10.1)	0.00	7.0 (3.5–9.9)	10.1 (4.8–17.1)	0.02	7.7 (4.0–12.7)	8.7 (4.7–13.1)	0.75
NT-pro BNP, pg/mL peak	205 (93–488.50)	205 (110–630)	0.69	166 (82–223)	283 (114–687)	0.02	172 (87–391)	273 (106–581)	0.23
hs Troponin I, ng/L peak	8.1 (3.4–21.3)	8.2 (4.2–18.7)	0.63	5.2 (2.6–17.1)	6.6 (4.1–21.6)	0.27	5.3 (3.6–18.9)	8.1 (4.0–52.4)	0.52
Fibrinogen, mg/dL peak	881 (725–109)	864 (739–1033)	0.71	880 (762–1019)	899 (740–1040)	0.8	826 (717–975)	886 (728–1040)	0.36

Abbreviations: CRP, C reactive protein; DLCOc, diffusing capacity for carbon monoxide corrected for hemoglobin; ESR, erythrocyte sedimentation rate; hs Troponin I, high-sensitivity cardiac troponin; IL-6, interleukin-6; IL-10, interleukin-10; LDH, lactate dehydrogenase; NLR, neutrophil-Lymphocyte Ratio; NT-pro BNP, N-terminal pro hormone B-type natriuretic peptide; PCRLA, post-COVID-19 radiological lung abnormalities; PDW, platelet distribution width; PE, pulmonary embolism; RDW, red cell distribution width. *, minimum value.

**Table 5:** Biomarkers of interstitial lung disease, inflammation, and coagulation collected on admission.

	Non-PE (n = 85)	PE (n = 56)	*p*	DLCOc ≥ 80% (n = 36)	DLCOc < 80% (n = 76)	*p*	No PCRLA (n = 43)	PCRLA (n = 53)	*p*
Plasminogen, ng/mL	580.8 (412–804.5)	535.3 (415.1–771)	0.83	599 (459.3–915.6)	525.1 (408.6–756.1)	0.32	694.9 (525.1–2046.6)	475.5 (239.3–617.3)	0.00
Protein C, µg/mL	13.9 (9.9–18.9)	14.8 (11.3–19.5)	0.88	13.9 (9.8–20.7)	13.9 (9.7–18.6)	0.60	13.8 (10.7–19)	14 (9.5–16.9)	0.77
P selectin, ng/mL	67.5 (29.9–213.1)	107.2 (40.3–230.9)	0.22	107.2 (26.1–213.1)	94 (32.9–213.1)	0.54	111.5 (38.5–227.7)	103.4 (32.8–213.1)	0.42
Sphingosine 1 phosphate receptor-1, ng/mL	2.1 (0.9–4.9)	1.6 (0.7–3.3)	0.06	4.15 (1.28–5)	1.3 (0.80–3.6)	0.04	2.34 (0.88–4.91)	1.36 (0.79–4.6)	0.11
VE-Cadherin, ng/mL	427.3 (322.5–601)	474.2 (322.8–648.6)	0.63	473.2 (364.4–692.1)	415.7 (304.5–578.2)	0.15	544.6 (327.7–728.9)	417 (288.7–551.7)	0.05
Galectin, ng/mL	175.7 (102.7–354.5)	210.7 (141.7–346.8)	0.30	168.5 (115.5–268.1)	205.7 (103.5–358.3)	0.53	204.4 (132.2–308.4)	178.5 (77–335.7)	0.32
Matrix metalloproteinase 7, ng/mL	7.4 (5.4–10.6)	6.3 (5.5–9.5)	0.39	6.6 (5.7–9.5)	6.5 (4.9–11)	0.88	6.5 (5.7–9.7)	7.2 (4.8–10.6)	0.95
Surfactant protein D, ng/mL	5.1 (0.7–17)	5.9 (2.3–14)	0.49	3.3 (0.5–8.1)	8 (3.2–24.5)	0.00	4.3 (0.4–11.46)	6.4 (2.2–26.1)	0.08
TNF-α, pg/mL	12.7 (4–16.9)	9.8 (5.7–16.2)	0.60	11.7 (4.4–17.1)	10.6 (5.4–16.7)	0.99	9.8 (3.8–16)	10.2 (5.7–16.2)	0.73
TNF-α receptor-1, ng/mL	1.8 (1.3–2.6)	1.6 (1.3–2.2)	0.47	1.5 (1.3–2.1)	1.8 (1.3–2.9)	0.31	1.5 (1.2–2)	1.7 (1.2–2.7)	0.22
TNF-α receptor-2, ng/mL	16.2 (9.7–45.3)	16.1 (9.6–28.8)	0.35	11.4 (8.7–26.7)	18.3 (11.1–42)	09	11.6 (8.4–24.1)	16.2 (11.5–28.9)	0.06
IL-1-β, pg/mL	1.6 (1–3.8)	1.63 (0.97–2.79)	0.52	1.4 (0.7–2.9)	1.6 (1–3.1)	0.2	1.2 (0.9–2.7)	1.8 (1.2–4.3)	0.02
CA15-3, U/mL	19.8 (8.8–68)	23.8 (10–81.4)	0.58	13.7 (8.8–38.1)	23.2 (10.5–97.1)	0.08	21.7 (13.3–68.2)	23.8 (9.9–88.2)	0.94
Macrophage inflammatory protein 4-α, ng/mL	8.8 (5.7–16.8)	10.3 (7.2–16.1)	0.46	9.4 (6.6–15.6)	9.6 (6.5–17)	0.94	12.1 (7.8–21.5)	8.5 (6.5–14.5)	0.05
Epidermal growth factor receptor, ng/mL	48.2 (40.4–64.9)	48.8 (40–61.7)	0.97	47.4 (38.1–55.6)	48.8 (39.1–62.4)	0.67	49.5 (38.8–57.3)	48.2 (40–61.9)	0.69

Plasma samples were collected during patient’s hospitalization and when CTPA was performed. Abbreviations: CA 15-3, carbohydrate antigen 15-3; DLCOc, diffusion capacity of carbon monoxide corrected for hemoglobin; IL, interleukin; PCRLA, post-COVID-19 radiological lung abnormalities; PE, pulmonary embolism; TNF, tumor necrosis factor; VE-Cadherin, vascular endothelial cadherin.

**Table 6:** PCRLA predictive score.

	Value
Age > 65 yrs.	
Yes	3
No	0
Minimum lymphocyte counts	
≤6.7 × 10^3^/µL	1
>6.7 × 10^3^/µL	0
IL-1β	
<1.2 pg/mL	0
≥1.2 pg/mL	2

## Data Availability

The data presented in this study are available for other researchers who meet the criteria for access to confidential data may request to gain access to the minimal data set underlying the results from the corresponding author. Besides, the researchers shall submit a methodological proposal. The data are not publicly available due to the applicable privacy regulation and Good Clinical Practices legislation. This dataset contains potentially identifying information, for example, age, BMI, and data of admission to the hospital leading to a unique subject in the dataset. Moreover, sharing individual participant data with third parties was not specifically included in the informed consent form of the study, and unrestricted diffusion of such data may pose a potential threat of revealing participants’ identities, as permanent data anonymization was not carried out.

## Supplementary Materials

The following supporting information can be downloaded at: https://www.mdpi.com/article/10.3390/ijms25137270/s1. References [75,76,77,78,79,80,81,82,83] are cited in the Supplementary Materials.
