# Supplementary material for: Cardiopulmonary Complications after Pulmonary Embolism in COVID-19
Source: Int J Mol Sci. 2024 Jul 2;25(13):7270. doi: 10.3390/ijms25137270 (PMC11242326; doi:10.3390/ijms25137270)
Supplement: Supplementary file 1 [file ijms-25-07270-s001.zip › ijms-3018769-supplementary.pdf]

Supplementary material

1. **MATERIAL AND METHODS**

Description of undertaken investigations

Epidemiological, demographic, clinical, and laboratory examinations were collected from all subjects at the time of admission. The data recorded included medical treatment during hospitalization, respiratory support, and clinical outcomes (acute respiratory failure, arrhythmia, ICU admission, or death). Cardiovascular disease included prior coronary heart disease (including myocardial infarction and angina) or chronic heart failure. Asthenia was defined as fatigue (easy tiredness and decreased ability to maintain performance), generalized weakness (anticipated feeling of difficulty in starting a certain activity) and/or mental fatigue (impaired mental concentration, memory loss and emotional lability).

The CURB-65 score was calculated[77]. The severity of the acute disease was determined according to the seven-category severity scale recommended by WHO[78], and the modified medical research council (mMRC) dyspnea scale was used to establish functional impairment due to dyspnea[79] at the follow-up.

Laboratory

As a secondary objective we compared baseline blood biomarkers according to the presence of respiratory and cardiovascular consequences.

We included COVID-19 patients with and without PE. Therefore, we decided to measure a group of biomarkers related with systemic inflammation, interstitial lung disease, acute lung injury, vascular permeability and endothelium, pulmonary hypertension, coagulation, and thrombosis:

- Inflammation biomarkers: CRP, IL-1-β, Galectin, TNF-α, TNF-α receptor-1, TNF-α receptor-2, Macrophage inflammatory protein 4-α, ferritin, LDH, ESR, D-dimer, IL-6, IL-10, RDW, NLR, PDW.

- Interstitial lung disease biomarkers: surfactant protein D, sphingosine 1 phosphate receptor-1, matrix metalloproteinase 7, CA15-3, macrophage inflammatory protein 4-α, Epidermal growth factor receptor, fibrinogen, RDW.

- Acute lung injury: sphingosine 1 phosphate receptor-1.

- Vascular permeability and endothelium function biomarkers: sphingosine 1 phosphate receptor-1, VE-Cadherin.

- Pulmonary hypertension: NT-pro BNP, hs Troponin I.

- Thrombosis: Galectin, D-dimer, fibrinogen, plasminogen, protein C, P selectin, PDW.

Laboratory data included complete blood count (Cell-Dyn Sapphire platform, Abbott Diagnostics, Lake forest, IL, United States), coagulation, and kidney and liver function tests collected upon admission. In addition, baseline, and peak values of the following biomarkers were analyzed in each patient: D-dimer (reported as D-dimer units (DDUs), ACL TOP 700, Instrumentation Laboratory), C-reactive protein (CRP), lactate dehydrogenase (LDH), erythrocyte sedimentation rate (ESR), ferritin, platelet and lymphocyte counts, neutrophil-to-lymphocyte ratio (NLR), red blood cell distribution width (RDW), platelet distribution width (PDW). In addition, high sensitive troponin I, interleukin-6 (IL-6), interleukin-10 (IL-10) (ELISA, R&D systems, Minneapolis, MN, USA), N-terminal pro hormone B-type natriuretic peptide (NT pro-BNP) (Test 1 THL Module, ALI FAX; Architect platform, Abbott Diagnostics), and fibrinogen were also measured. Blood gas analyses were performed on the GEM 4000 platform (Werfen, Barcelona, Spain).

D-dimer was considered elevated when >230 ng/ml for the assays expressing results as DDU. In the case of patients over 50 years of age, the age-adjusted D-dimer cutoff point (age x 5) was applied.

Pulmonary function tests

Spirometry and lung diffusing capacity for carbon monoxide (DLCO) measurements were performed by experienced technicians (MasterLab Pro; Erich Jaeger GmbH; Höchberg, Germany) according to the European Respiratory Society and American Thoracic Society recommendations[80,81].

Six-Minute Walking Test (6MWT)

A 6MWT was performed according to recommended guidelines with baseline and after-exercise oxyhemoglobin saturation (SpO_2) by pulse oximetry on index fingers[82]. The distance walked during 6MWT was measured and compared with 6MWT predictive values according to the reference values[83].

Echocardiography

Standard 2D Doppler transthoracic echocardiography was performed according to recommendations of the American Society of Echocardiography[84]. All echocardiography studies were conducted by certified and experienced cardiologists (GE Vivid E9 with GE M5S-D probe; 1.5–4.5 MHz of frequency range with a posterior off-line analysis with software EchoPAC Version 113 (GE Healthcare). Echocardiographic probability of pulmonary hypertension was obtained according to recommendations of the 2022 ESC/ERS Guidelines for the diagnosis and treatment of pulmonary hypertension[85].

2. RESULTS

Supplementary table S1. Clinical characteristics at medium-term (all patients) and according to outcome (with and without pulmonary thrombosis) during

	All (n= 141)	Non-PE (n= 85)	PE (n= 56)	p value
Dyspnea, n (%)	44 (37.9)	20 (31.7)	24 (45.3)	0.13
mMRC 0	1 (2.4)	0 (0)	1 (4.5)	
mMRC 1	23 (56.1)	11 (57.9)	12 (54.4)	
mMRC 2	14 (34.1)	8 (42.1)	6 (27.3)	
mMRC 3	3 (7.3)	0 (0)	3 (13.6)	
Cough, n (%)	12 (10.8)	6 (10)	6 (11.8)	0.87
Asthenia, n (%)	27 (24.5)	15 (25)	12 (24)	0.68
Headache, n (%)	11 (10.4)	9 (15.3)	2 (4.3)	0.11
Arthralgias, n (%)	16 (15.2)	9 (15.8)	7 (14.6)	0.74
Dysphonia, n (%)	3 (2.8)	2 (3.4)	1 (2)	1.00
Post COVID-19 condition, n (%)	5 (4.4)	5 (7.9)	0 (0)	0.07

Abbreviations: mMRC: Modified Medical Research Council; PE, pulmonary embolism.

Supplementary table S2. Radiological findings at medium-term (all patients) and according to outcome (with and without pulmonary thrombosis) during hospitalization.

	All (n= 96)	Non-PE (n= 52)	PE (n= 44)	p value
Pulmonary thrombosis, n (%)	7 (7.3)	2 (3.8)	5 (11.4)	0.34
Chronic thromboembolic disease, n (%)	2 (2.1)	0 (0)	2 (4.3)	0.28
PCRLA	53 (55.2)	27 (51,9)	26 (59.1)	0.48
Ground-glass opacity, n (%)	28 (29.2)	14 (26.9)	14 (31.8)	0.59
Fibrotic bands, n (%)	24 (25)	9 (17.3)	15 (34.1)	0.06
Septal thickening	24 (25)	13 (25)	11 (25)	1.00
Bronchiectasis, n (%)	22 (22.9)	11 (21.2)	11 (25)	0.66
Alveolar opacities, n (%)	2 (2.1)	1 (1.9)	1 (2.3)	1.00
Crazy-paving, n (%)	2 (2.1)	2 (3.8)	0 (0)	0.49
Pleural thickening, n (%)	7 (7.3)	4 (7.7)	3 (6.8)	1.00
Pleural effusion, n (%)	3 (3.1)	0 (0)	3 (6.8)	0.09

Abbreviations: PCRLA, post-COVID-19 radiological lung abnormalities; PE, pulmonary embolism.

Supplementary table S3. Echocardiography findings at medium-term (all patients) and according to outcome (with and without pulmonary thrombosis)

	All (n= 113)	Non-PE (n=67)	PE (n=46)	p value
LVEF, %	63.78 ± 6.54	63.58 ± 7.0	64.09 ± 5.8	0.69
TAPSE, mm	22.34 ± 3.17	22.42 ± 3.3	22.18 ± 2.9	0.77
Tricuspid regurgitation pressure gradient, mmHg	22 (20-25)	21 (19-25)	23 (21-25)	0.51
Pulmonary artery pressure systolic, mmHg	27 (25-30)	26 (24-30)	28 (27-30)	0.12
Pulmonary hypertension, intermediate-high probability, n (%)	4 (3.5)	1 (1.5)	3 (6.5)	0.3

Abbreviations: LVEF, left ventricular ejection fraction; PE, pulmonary embolism; TAPSE, tricuspid annular plane systolic excursion.

Supplementary Table S4. Factors associated with DLCOc < 80%.

	Odds ratio	95% CI	p value
Age >65 yrs.	2.67	1.13-6.3	**0.02**
WHO severity classification	3	1.3-6.9	**0.01**
CURB-65	2.39	1.33-4.27	**0.00**
Length of hospital stay, days	1.07	1.03-1.12	**0.00**
Length of ICU stay, days	1.06	1.01-1.11	**0.02**
OTI length, days	1.10	1.01-1.18	**0.02**
High flow oxygen	3.76	1.31-10.84	**0.01**
Maximum FiO_2	6.36	1.86-21.69	**0.00**
Baseline CRP	1.03	1.00-1.06	**0.03**
Human surfactant associated protein D	1.06	1.01-1.12	**0.02**
Peak red cell distribution width	1.45	1.09-1.94	**0.01**
Peak IL-10	1.04	1.01-1.08	**0.01**

Abbreviations: CRP, C reactive protein; FiO_2, fraction of inspired oxygen; ICU, intensive care unit; IL-10, interleukin-10; OTI, orotracheal intubation; WHO, world health organization.

Supplementary Table S5. Factors associated with PCRLA.

	Odds ratio	95% CI	p value
Age >65 yrs.	4.43	1.84-10.67	**0.00**
Invasive ventilation	3.62	1.42-9.26	**0.01**
Length of ICU stay	1.06	1.01-1.10	**0.02**
Prone position	6	1.26-28.5	**0.01**
Corticosteroids, bolus	2.62	1.01-6.79	**0.04**
CURB-65	1.98	1.08-3.63	**0.03**
Maximum FiO_2	4.78	1.36-16.71	**0.01**
Peak total bilirubin	3.4	1.24-9.36	**0.02**
Minimum lymphocyte counts	0.33	0.11-0.99	**0.04**
IL-1β	1.34	1.03-1.75	**0.03**

Abbreviations: FiO_2, fraction of inspired oxygen; ICU, intensive care unit; IL-10, interleukin-10.

Supplementary Table S6. Independent factors associated with PCRLA.

	Unstandardized regression coefficients		Standardized regression coefficients	95% CI for B		
	B	SE	B	Lower limit	Upper limit	P value
Age >65 yrs.	1.787	0.585	5.97	1.895	18.807	0.002
Minimum lymphocyte counts	1.79	0.558	5.992	2.007	17.886	0.001
IL-1β	1.896	0.605	6.658	2.034	21.792	0.002

Abbreviations: IL-1β, interleukin-1β.

Supplementary Table S7. Sensitivity, specificity, PPV and NPV of different cut-off points for PCRLA predictive score

	Sensitivity, %	Specificity, %	PPV, %	NPV, %	FPR, %	FNR,
Score ≥ 1	96.2	14	58	75	86	3.8
Score ≥ 2	96.2	32.6	63.8	87.5	67.4	3.8
Score ≥ 3	83	65.1	74.6	75.7	34.9	17
Score ≥ 4	54.7	83.7	80.6	60	16.3	45.3
Score ≥ 5	45.3	88.4	82.8	56.7	11.6	54.7
Score = 6	28.3	97.7	93.8	52.5	2.3	71.7

Abbreviations: FNR, false negative rate; FPR, false positive rate; PPV, positive predictive values; NPV, negative predicted values.

Supplementary Table S8. Baseline anthropometric and clinical characteristics of patients admitted because of COVID-19 pneumonia. Number of missing data per variable

	All (n= 141)
Age, n (%)	0 (0)
Age >65 yrs, n (%)	0 (0)
BMI, n (%)	2 (1.4)
Smoking status, n (%)	0 (0)
Current smoker, n (%)	0 (0)
Former smoker, n (%)	0 (0)
Smoking, n (%)	0 (0)
Hypertension, n (%)	0 (0)
Diabetes mellitus, n (%)	0 (0)
Cardiovascular disease, n (%)	0 (0)
Cerebrovascular disease, n (%)	0 (0)
Chronic kidney disease, n (%)	0 (0)
COPD, n (%)	0 (0)
Asthma, n (%)	0 (0)
**COVID-19 Admission**	
Physical examination, n (%)	
Respiratory rate, n (%)	25 (17.7)
Heart rate, n (%)	2 (1.4)
Systolic BP, n (%)	1 (0.7)
Diastolic BP, n (%)	1 (0.7)
Temperature, n (%)	1 (0.7)
CURB 65	26 (18.4)
WHO severity classification	0 (0)
Treatment in hospital	
High flow oxygen, n (%)	1 (0.7)
Non-Invasive ventilation, n (%)	0 (0)
Invasive ventilation, n (%)	1 (0.7)
Prone position, n (%)	0 (0)
Azithromycin, n (%)	0 (0)
Hydroxychloroquine, n (%)	0 (0)
Lopinavir/ritonavir, n (%)	0 (0)
Remdesivir, n (%)	1 (0.7)
IFNb, n (%)	0 (0)
Tocilizumab, n (%)	0 (0)
Systemic Corticosteroids, n (%)	0 (0)
ICU, n (%)	0 (0)

Abbreviations: BMI, body mass index; BP, blood pressure; COPD, chronic obstructive pulmonary disease; ICU, intensive care unit; IFNb, interferon beta; WHO (world health organization) severity.

Supplementary Table S9. Pulmonary function and six-minute walking test at medium-term follow-up. **Number of missing data per variable**

	All (n= 119)
FVC, n (%)	1 (0.8)
FEV1, n (%)	1 (0.8)
FEV1/FVC, n (%)	1 (0.8)
DLCOc, n (%)	8 (6.7)
KCOc, n (%)	8 (6.7)
Walking distance, n (%)	28 (23.5)
Walking distance, n (%)	28 (23.5)
Resting oxygen saturation, n (%)	28 (23.5)
End-exercise oxygen saturation, n (%)	28 (23.5)
Lowest oxygen saturation, n (%)	29 (24.4)

Abbreviations: DLCOc, Diffusing capacity for carbon monoxide corrected for hemoglobin; FEV1, forced expiratory volume in the first second; FVC, Forced vital capacity; PE, Pulmonary embolism.

Supplementary Table S10. Baseline and medium-term follow-up laboratory data. Number of missing data per variable

	All (n= 141)
**Blood count, baseline**	
Hemoglobin, n (%)	0 (0)
Leucocyte count, n (%)	0 (0)
Lymphocyte count, n (%)	0 (0)
Neutrophil counts, n (%)	0 (0)
**Biochemical profile, baseline, n (%)**	
Glucose, n (%)	0 (0)
ALT, n (%)	1 (0.7)
Urea, n (%)	0 (0)
Creatinine, n (%)	0 (0)
Sodium, n (%)	0 (0)
Potassium, n (%)	0 (0)
Cholesterol, n (%)	11 (7.8)
Triglyceride, n (%)	11 (7.8)
**Coagulation function**	
PT, n (%)	1 (0.7)
INR, n (%)	1 (0.7)
Fibrinogen, n (%)	6 (4.3)
**Arterial blood test**	
PaO_2/FiO_2 ratio, n (%)	6 (4.3)
pH, n (%)	8 (5.7)
PaO_2, n (%)	6 (4.3)
$PaCO_2$, n (%)	7 (5)
**Follow-up laboratory findings**	
Hemoglobin, n (%)	9 (6.4)
Leukocyte count, n (%)	9 (6.4)
Neutrophil count, n (%)	9 (6.4)
Lymphocyte count, n (%)	9 (6.4)
Platelet count, n (%)	9 (6.4)
ERS, n (%)	15 (10.6)
D-dimer, n (%)	21 (14.9)
Elevated D-dimer, n (%)	21 (14.9)
CRP, n (%)	13 (9.2)
Ferritin, n (%)	14 (9.9)
LDH, n (%)	21 (14.9)
NT-pro-BNP, n (%)	14 (9.9)

Abbreviations: ALT, alanine transaminase; CPR, C reactive protein; FiO2, fraction of inspired oxygen; INR, international normalized ratio; LDH, lactate dehydrogenase; NT-pro-BNP, N-terminal pro hormone B-type natriuretic peptide; PaO_2, arterial partial pressure of oxygen; $PaCO_2$, arterial partial pressure of carbon dioxide; PE, pulmonary embolism; PT%, prothrombin time.

Supplementary Table S11. Inflammatory and thrombotic biomarkers on admission according to outcome. Number of missing data per variable

	PE comparisons (n=141)	DLCOc comparisons (n=112)	PCRLA comparisons (n=96)
LDH			
Baseline, n (%)	1 (0.7)	1 (0.9)	0 (0)
Peak, n (%)	0 (0)	0 (0)	0 (0)
CRP			
Baseline, n (%)	1 (0.7)	0 (0)	1 (1)
Peak, n (%)	0 (0)	0 (0)	0 (0)
ESR			
Baseline, n (%)	14 (9.9)	10 (8.9)	7(13.2)
Peak, n (%)	5 (8.7)	5 (4.5)	5 (9.4)
D-dimer			
Baseline, n (%)	0 (0)	0 (0)	0 (0)
Peak, n (%)	0 (0)	0 (0)	0 (0)
Ferritin			
Baseline, n (%)	2 (1.4)	2 (1.8)	1 (1.9+)
Peak, n (%)	1 (0.7)	1 (0.9)	1 (1.9)
Platelet count			
Baseline, n (%)	0 (0)	0 (0)	0 (0)
Peak, n (%)	0 (0)	0 (0)	0 (0)
Lymphocyte counts			
Baseline, n (%)	0 (0)	0 (0)	0 (0)
Peak*, n (%)	0 (0)	0 (0)	0 (0)
NLR			
Baseline, n (%)	0 (0)	0 (0)	0 (0)
Peak, n (%)	0 (0)	0 (0)	0 (0)
RDW, %			
Baseline, n (%)	0 (0)	0 (0)	0 (0)
Peak, n (%)	0 (0)	0 (0)	0 (0)
PDW, %			
Baseline, n (%)	0 (0)	0 (0)	0 (0)
Peak, n (%)	0 (0)	0 (0)	0 (0)
IL-6, n (%)	31 (22)	24 (21.4)	13 (24.4)
IL-10, n (%)	40 (28.4)	28 (25)	16 (30.2)
NT-pro BNP, n (%)	17 (12.1)	10 (8.9)	6 (11.3)
hs Troponin I, n (%)	69 (48.9)	51 (55.5)	19 (35.8)
Fibrinogen, n (%)	0 (0)	0 (0)	0 (0)

Abbreviations: CRP, C reactive protein; DLCOc, diffusing capacity for carbon monoxide corrected for hemoglobin; ESR, erythrocyte sedimentation rate; hs Troponin I, high-sensitivity cardiac troponin; IL-6, interleukin-6; IL-10, interleukin-10; LDH, lactate dehydrogenase; NRL, neutrophil-Lymphocyte Ratio;NT-proBNP, N-terminal pro hormone B-type natriuretic peptide; PCRLA, post-COVID-19 radiological lung abnormalities; PDW, platelet distribution width; PE, pulmonary embolism; RDW, red cell distribution width.

Supplementary Table S12. Biomarkers of interstitial lung disease, inflammation, and coagulation collected on admission. Number of missing data per variable

	PE comparisons (n=141)	DLCOc comparisons (n=112)	PCRLA comparisons (n=96)
Plasminogen, n (%)	18 (12.8)	12 (10.7)	10 (10.4)
Protein C, n (%)	19 (13.5)	13 (11.6)	10 (10.4)
P selectin, n (%)	18 (12.8)	12 (10.7)	10 (10.4)
Sphingosine 1 phosphate receptor-1, n (%)	21 (14.9)	15 (13.4)	11 (11.5)
VE-Cadherin, n (%)	18 (12.8)	12 (10.7)	10 (10.4)
Galectin, n (%)	18 (12.8)	12 (10.7)	10 (10.4)

**Matrix metalloproteinase 7, n (%)**	18 (12.8)	12 (10.7)	10 (10.4)
**Surfactant protein D, n (%)**	25 (17.7)	18 (16.1)	13 (13.5)
**TNF-α, n (%)**	22 (15.6)	16 (14.3)	12 (12.5)
**TNF-α receptor-1, n (%)**	18 (12.8)	12 (10.7)	10 (10.4)
**TNF-α receptor-2, n (%)**	18 (12.8)	12 (10.7)	10 (10.4)
**IL-1-β, n (%)**	22 (15.6)	16 (14.3)	12 (12.5)
**CA15-3, n (%)**	19 (13.5)	13 (11.6)	11 (11.5)
**Macrophage inflammatory protein 4-α, n (%)**	18 (12.8)	12 (10.7)	10 (10.4)
**Epidermal growth factor receptor, n (%)**	22 (15.6)	16 (14.3)	12 (12.5)

Plasma samples were collected during patient's hospitalization and when CTPA was performed. Abbreviations: CA 15-3, carbohydrate antigen 15-3; DLCOc, diffusion capacity of carbon monoxide corrected for hemoglobin; IL, interleukin; PCRLA, post-COVID-19 radiological lung abnormalities; PTPE, pulmonary embolism; TNF, tumor necrosis factor; VE-Cadherin, vascular endothelial cadherin.

Supplementary table S 13. Clinical characteristics at medium-term (all patients) and according to outcome (with and without pulmonary thrombosis) during hospitalization. Number of missing data per variable

	All (n= 141)
Dyspnea, n (%)	25 (17.7)
mMRC, n (%)	3 (6.8)
Cough, n (%)	30 (21.3)
Asthenia, n (%)	31 (22)
Headache, n (%)	35 (24.8)
Arthralgias, n (%)	36 (25.5)
Dysphonia, n (%)	34 (24.1)
Post COVID-19 condition, n (%)	27 (19.1)

Abbreviations: mMRC: Modified Medical Research Council; PE, pulmonary embolism.

Supplementary figure S1: Area under the ROC curve (AUC) of score for PCRLA.

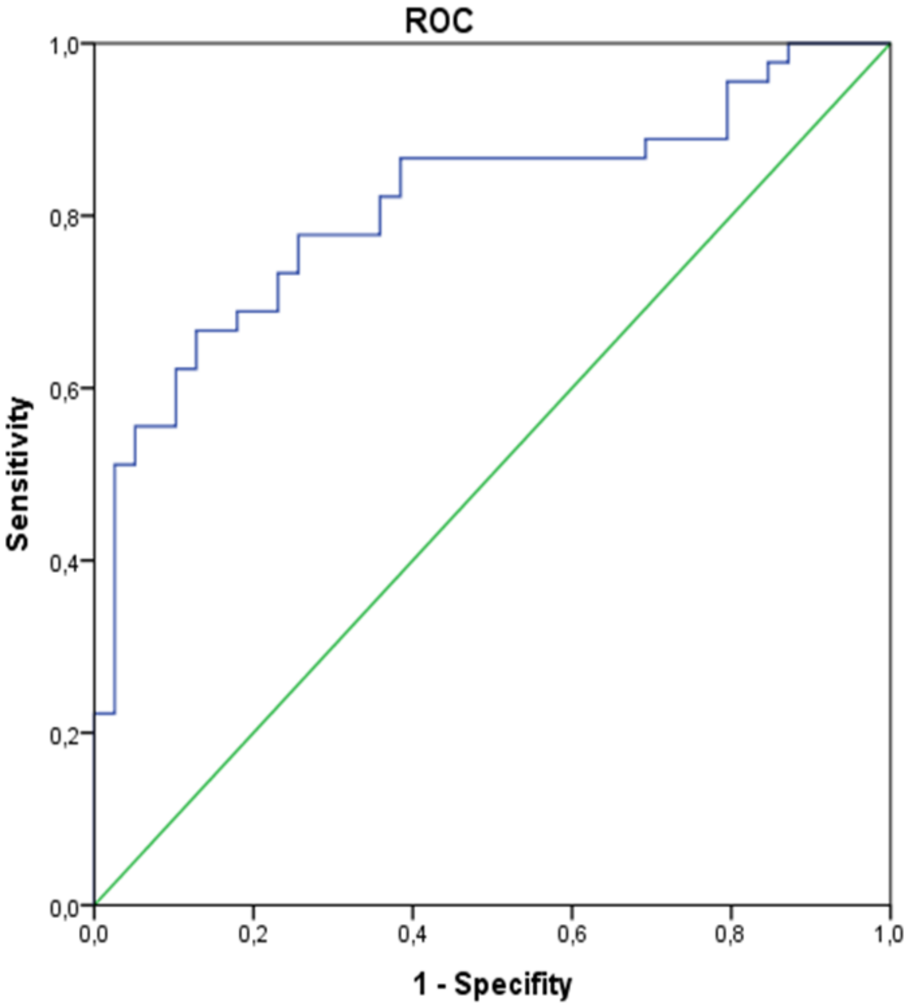

3. References

[1] Lim, W. S., Van Der Eerden, M. M., Laing, R., Boersma, W. G., Karalus, N., Town, G. I., and Lewis, S. A., "Defining Community Acquired Pneumonia Severity on Presentation to Hospital: An International Derivation and Validation Study," 2003. https://doi.org/10.1136/thorax.58.5.377

[2] World Health Organization (WHO), "World Health Organization Clinical Management of Severe Acute Respiratory Infection When Novel Coronavirus (NCoV) Infection Is Suspected: Interim Guidance," Feb 26 2020. Retrieved 14 December 2023. https://www.Who.Int/publications-detail/clinical-management-of-severe-acute-respiratory-infection-when-novel-coronavirus-(ncov)-infection-is-suspected

[3] Williams N, "The MRC Breathlessness Scale," *Occup Med (Lond)*, Vol. 67, No. 6, 2017, pp. 496–497. https://doi.org/10.1093/occmed/kqx086

[4] Graham BL, Steenbruggen I, Miller MR, Barjaktarevic IZ, Cooper BG, Hall GL, Hallstrand TS, Kaminsky DA, McCarthy K, McCormack MC, Oropez CE, Rosenfeld M, Stanojevic S, Swanney MP, and Thompson BR, "Standardization of Spirometry 2019 Update," *An Official American Thoracic Society and European Respiratory Society Technical Statement. Am J Respir Crit Care Med.*, Vol. 200, No. 8, 2019. https://doi.org/10.1164/rccm.201908-1590ST

[5] Graham BL, Brusasco V, Burgos F, Cooper BG, Jensen R, Kendrick A, MacIntyre NR, Thompson BR, and Wanger J, "2017 ERS/ATS Standards for Single-Breath Carbon Monoxide Uptake in the Lung," *Eur Respir J.* , Vol. 49, No. 1, 2017. https://doi.org/10.1183/13993003.00016-2016

[6] Singh SJ, Puhan MA, Andrianopoulos V, Hernandes NA, Mitchell KE, Hill CJ, Lee AL, Camillo CA, Troosters T, Spruit M, Carlin BW, Wanger J, Pepin V, Saey D, Pitta F, Kaminsky DA, McCormack MC, MacIntyre N, Culver BH, Sciurba FC, Revill SM, Delafosse V, and Holland AE, " An Official Systematic Review of the European Respiratory Society/American Thoracic Society: Measurement Properties of Field Walking Tests in Chronic Respiratory Disease ," *Eur Respir J*, Vol. 44, No. 6, 2014, pp. 1447–1448. https://doi.org/10.1183/09031936.00150414

[7] Enright PL, and Sherrill DL., "Reference Equations for the Six-Minute Walk in Healthy Adults ," *Am J Respir Crit Care Med*, Vol. 158, No. 5, 1998, pp. 1387–1387. https://doi.org/10.1164/ajrccm.158.5.9710086

[8] Lang, R. M., Badano, L. P., Victor, M. A., Afilalo, J., Armstrong, A., Ernande, L., Flachskampf, F. A., Foster, E., Goldstein, S. A., Kuznetsova, T., Lancellotti, P., Muraru,

D., Picard, M. H., Retzschel, E. R., Rudski, L., Spencer, K. T., Tsang, W., and Voigt, J. U., "Recommendations for Cardiac Chamber Quantification by Echocardiography in Adults: An Update from the American Society of Echocardiography and the European Association of Cardiovascular Imaging," *Journal of the American Society of Echocardiography*, Vol. 28, No. 1, 2015, pp. 1-39.e14. https://doi.org/10.1016/j.echo.2014.10.003

[9] Humbert, M., Kovacs, G., Hoeper, M. M., Badagliacca, R., Berger, R. M. F., Brida, M., Carlsen, J., Coats, A. J. S., Escribano-Subias, P., Ferrari, P., Ferreira, D. S., Ghofrani, H. A., Giannakoulas, G., Kiely, D. G., Mayer, E., Meszaros, G., Nagavci, B., Olsson, K. M., Pepke-Zaba, J., Quint, J. K., Rådegran, G., Simonneau, G., Sitbon, O., Tonia, T., Toshner, M., Vachiery, J. L., Vonk Noordegraaf, A., Delcroix, M., Rosenkranz, S., Schwerzmann, M., Dinh-Xuan, A. T., Bush, A., Abdelhamid, M., Aboyans, V., Arbustini, E., Asteggiano, R., Barberà, J. A., Beghetti, M., Čelutkienė, J., Cikes, M., Condliffe, R., de Man, F., Falk, V., Fauchier, L., Gaine, S., Galié, N., Gin-Sing, W., Granton, J., Grünig, E., Hassoun, P. M., Hellemons, M., Jaarsma, T., Kjellström, B., Klok, F. A., Konradi, A., Koskinas, K. C., Kotecha, D., Lang, I., Lewis, B. S., Linhart, A., Lip, G. Y. H., Løchen, M. L., Mathioudakis, A. G., Mindham, R., Moledina, S., Naeije, R., Nielsen, J. C., Olschewski, H., Opitz, I., Petersen, S. E., Prescott, E., Rakisheva, A., Reis, A., Ristić, A. D., Roche, N., Rodrigues, R., Selton-Suty, C., Souza, R., Swift, A. J., Touyz, R. M., Ulrich, S., Wilkins, M. R., Wort, S. J., Krim, M., Hayrapetyan, H., Musayev, O., Lazareva, I., Sokolović, Š., Velchev, V., Michaloliakos, I., Jansa, P., Mellemkjær, S., Hassan, A., Anton, L., Pentikäinen, M., Meneveau, N., Tsverava, M., Lankeit, M., Manginas, A., Hizoh, I., Maher, V., Hirsch, R., Mukarov, M. A., Ibrahimi, P., Talant, S., Rudzitis, A., Kiwan, G., Gumbienė, L., Codreanu, A., Micallef, J., Vataman, E., Bulatovic, N., Chraibi, S., Post, M. C., Kostovska, E. S., Andreassen, A. K., Kurzyna, M., Plácido, R., Coman, I. M., Vasiltseva, O., Zavatta, M., Šimkova, I., Poglajen, G., Lázaro Salvador, M., Söderberg, S., Marjeh, M. Y. B., Ouarda, F., Mutlu, B., Sirenko, Y., Coghlan, J. G., Abdullaev, T., Baigent, C., Antoniou, S., Arbelo, E., Baumbach, A., Borger, M. A., Collet, J. P., Gale, C. P., Halvorsen, S., Iung, B., Landmesser, U., Sitges, M., Morgan, R. L., and Sivakumaran, K., "2022 ESC/ERS Guidelines for the Diagnosis and Treatment of Pulmonary Hypertension," *European Heart Journal*. 38. Volume 43, 3618–3731. https://doi.org/10.1093/eurheartj/ehac237
